# Impact of Elevated Temperatures on the Integrity of PIR-Core Roof Sandwich Panel Connections

**DOI:** 10.3390/ma19010064

**Published:** 2025-12-23

**Authors:** Anita Pawlak-Jakubowska, Paweł Krause, Artur Miros, Jiří Teslík, Michał Sitek

**Affiliations:** 1 Faculty of Civil Engineering, Silesian University of Technology, 44-100 Gliwice, Poland; pawel.krause@polsl.pl; 2 Łukasiewicz Research Network—Warsaw Institute of Technology, Center of New Technologies for Construction, 01-796 Warsaw, Poland; artur.miros@wit.lukasiewicz.gov.pl; 3 Faculty of Civil Engineering, VSB—Technical University of Ostrava, 708 33 Ostrava-Poruba, Czech Republic; jiri.teslik@vsb.cz; 4 Faculty of Architecture, Silesian University of Technology, 44-100 Gliwice, Poland

**Keywords:** sandwich panel, PIR foam core, joint, fire resistance, temperature distribution, heat flux, FEM modeling

## Abstract

The article presents an evaluation of the behavior of joints in roof sandwich panels with a PIR foam core, under conditions of short-term exposure to high temperatures. The aim of the study was to analyze the temperature field distribution within the joint and to investigate how a slight unsealing of the joint by approximately 3 mm affects the thermal insulation. Experimental studies using thermography and numerical analysis made it possible to determine the impact of slight joint gaps on the temperature distribution on the surface of the panels. The temperature difference between the reference areas and the areas exposed to fire did not exceed 1 °C. Using the finite element method, numerical models of joints with a gap of up to 6.81 mm were created. The thermal transmittance values ranged from 0.187 to 0.196 W/(m^2^·K), and heat flux density at a 102 °C difference from 19.237 to 20 W/m^2^. Even with slight panel separation, the joint still meets the requirements for insulation and fire resistance. Short-term exposure to 100 °C caused no damage, except from ~1 mm local PIR foam melting, which is harmless. Proper roof installation, in accordance with the manufacturer’s guidelines ensured the tightness and thermal resistance of the roof.

## 1. Introduction

The design and construction of buildings, including roof partitions made of sandwich panels, is based in particular on meeting the requirements for structural and operational safety, energy efficiency, environmental protection and fire safety. In order to achieve fire resistance of a building, it is essential to use construction products with the appropriate fire resistance class and solutions that prevent the spread of fire. The member states of the European Union use harmonized EU regulations, as well as national standards and regulations, to meet the fire protection requirements for buildings. The basic legal act is Regulation (EU) No 305/2011 of the European Parliament and of the Council [1], which lays down the conditions for the marketing of construction products that meet fire safety requirements. The main standards defining the classification of the behavior of construction products in fire conditions and the duration of fire resistance are EN 13501-1 [2] and EN 13501-2 [3]. In Poland, the basic legal act regulating the requirements for the design and construction of buildings is the Construction Law [4]. In addition to European standards [2,3], detailed fire safety requirements are set out in the Regulation of the Minister of Infrastructure on the technical conditions to be met by buildings and their location [5].

**Figure 1:**
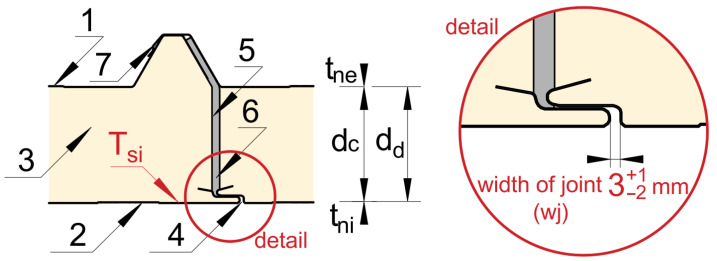
Schematic diagram of the joint between adjacent panels: (1) external facing, (2) internal facing, (3) PIR foam core, (4) lower part of the joint—width of the longitudinal joint between the roof panels, (5) film to prevent gas diffusion and water vapor infiltration into the core, (6) polyurethane gasket over the entire length of the joint, and (7) butyl mass or tape. Here, *T_si_* is the internal surface temperature of the facing and *wj* is the width of the lower part of the joint.

Structural efficiency, ease of erection, mass-production capabilities and thermal-insulation qualities of sandwich panels are commonly used in the construction of industrial facilities [6,7,8,9,10,11]. Their major advantages include their high strength and stiffness-to-weight ratio, their resistance to corrosion, a low thermal conductivity [12,13,14] and, in many cases, high fire resistance [15]. Self-supporting sandwich panels with a PIR foam core are successfully used for roof partitions in industrial construction. They consist of metal facings, both external and internal, between which there is a core made of thermal insulation material, most often in the form of, for example, non-flammable mineral wool MW, self-extinguishing EPS polystyrene or PUR polyurethane foam or PIR polyisocyanurate foam (Figure 1). ‘Polyurethane foam (PUR) and polyisocyanurate foam (PIR) are examples of most commonly applied thermal insulation materials in building construction‘ [16] (p. 1). PIR foam is a material that achieves a very low thermal conductivity coefficient *λ* in the range from 0.019 to 0.022 W/(m·K), according to the manufacturers, it remains stable up to 200 °C [17,18,19,20]. It is currently the best available thermal insulation material for use as a core. The core thickness *d_c_* of the panels ranges from 40 to 200 mm, and the effective (modular) width of a single panel ranges from 1000 to 1100 mm. The maximum length of the panel depends, among other things, on the selected color scheme, the method of fastening and the adopted static layout (related to snow and wind loads), and ranges from 2000 to 18,500 mm. In Poland, sandwich panels are manufactured in accordance with the harmonized standard EN 14509:2013 ‘Self-supporting insulating and structural sandwich panels with double-sided metal cladding’ [21].

The requirements for the fire resistance class of sandwich panel roof partitions are set out in paragraph 216 of the regulations entitled ‘Requirements for the fire resistance class of building elements’ [5]. For roofs, the fire resistance class of the supporting structure under the panels and the panel covering itself must be considered. Depending on the fire resistance class of the building, from A1 (non-combustible) to F (analyzed in minutes) [2,5], the fire resistance REI where load bearing (R), fire integrity (E), and fire insulation (I) can be determined [3,5]. Mainly, roofs made of sandwich panels have a fire resistance of REI 30. With regard to the reaction to fire class, these products are most often classified as B-s1,d0 or B-s2,d0, which qualifies them as non-combustible materials (in accordance with Appendix 3 of the regulations [5]). In terms of external fire resistance, they have a B_ROOF_ (t1) class, determined in accordance with the test method contained in the CEN/TS 1187:2014 standard ‘Methods for testing the external fire resistance of roofs’ [22], which means that they are non-fire-spreading products (NRO).

The fire resistance of the sandwich panels is closely related to the characteristics of the core material, especially its thermal decomposition temperature [23]. Thanks to its closed-cell structure and ablative properties at high temperatures and during a fire, the PIR foam thermal insulation hardens to form a fire-resistant layer of chars. The depth of charring gradually increases with the duration of the fire. The process stops when the fire is extinguished or the source of the fire is removed [24]. The resulting layer of charred material protects the deeper layers of foam from the effects of temperature, thus delaying its degradation. Thanks to the use of a larger amount of isocyanate in the production of the PIR foam, it is characterized by a greater resistance of polymer bonds to temperatures ranging from 300 to 325 °C [25]. Many researchers have analyzed the fire behavior of MW, PUR and PIR foam in their work [26,27,28,29,30,31].

The authors of articles [32,33,34] carried out experiments on fire spread in partition systems made of sandwich panels with a PIR foam core in their work. Murillo et al. focused on identifying the fire reaction behavior of five PIR foam core sandwich panels with vertical joints [35]. These were mainly carried out in laboratory and controlled environmental conditions. ‘There are few studies that investigate the behavior of this type of panels exposed to fire on a full scale’ [35] (p. 9489).

A study by Pyl et al. [36] constructed a full size, steel frame, industrial building for a fire experiment. The building’s cladding consisted of stone wool sandwich panels, whereas the roof was made of PIR sandwich panels, the behavior of the structure at elevated temperatures was analyzed. Researchers mainly focus on determining the fire response of the entire panel in laboratory tests and the experiments conducted lead in most cases to the destruction of the sandwich panels. In turn, Ablaoui et al. [37] proposed that presents a complex numerical model for modeling the behavior of a sandwich panel wall exposed to fire temperatures.

Currently, there are few studies dealing with the issue of analyzing the air-tightness of sandwich panel joints when the panel is exposed to high temperatures. A study by M. Garifullin et al. analyze the strength and stiffness of joints at increased temperatures. It has been established that ‘the core material seems to influence the rate of degradation of the connection resistance and stiffness at elevated temperatures, with PIR core showing greater degradation than MW’ [38] (p. 14).

In contrast, Wang and Foster’s evaluate the joint of sandwich façade panels given fire tests. It was determined that ‘a large gap forms in the joints of sandwich panels due to ablation of the exposed PIR core at high temperatures, resulting in temperature increases seen on the unexposed sides of the joints of the panel assemblies’ [39] (p. 14). In the analyses, the joint was subjected to direct fire which resulted in significant failure. The focus was on laboratory tests and the joint was not tested for roof panels.

Roof sandwich panels are a material commonly used in industrial hall buildings. This article discusses the issue of the resistance to high temperatures of partitions constructed from sandwich panels with a PIR foam core. The novelty of this research consists of the analysis of the joint area between adjacent roof panels in an existing building. The partition was not subjected to direct fire exposure, it was affected by the high temperatures generated by the fire. This issue, which has been overlooked in the literature so far, is a significant contribution to the knowledge of fire resistance of sandwich panel structures and is crucial for user safety and structural durability. In contrast to previous studies, which focused on laboratory tests of wall connections exposed to fire, this research is the first to investigate the impact of high temperatures on joint integrity under conditions of indirect fire influence.

Exposure to high temperatures can damage the structure of the partition components and cause changes in the physical properties of the materials. The formation of damage, e.g., gaps, cracks, deformations, may increase heat flow and lead to a deterioration of thermal insulation properties, in particular at the point where two panels are joined together. In roof structures made of sandwich panels, the joint is a minor thermal bridge [40] and is particularly vulnerable to damage and leaks. These may result from installation errors, operation, and use of the structure, e.g., snow and rain loads, but also from thermal loads or material aging processes. In exceptional situations, high temperatures can affect partitions, which may be caused by the operation of machinery, equipment, metallurgical furnaces, or even a fire. These structures require regular diagnostics of joints, e.g., using thermal imaging to identify possible irregularities.

In their considerations, the authors focused on identifying damage or deformation of the facing and core, particularly in the area of the panel joint, as a result of short-term exposure of the partition to high temperatures. This research aims to assess the temperature distribution *T_si_* (detailed in Figure 1) on the inner facing of the plates within the joint.

The identification of any anomalies in the distribution may indicate possible damage, leaks, and irregularities inside the joint that affect the safety, thermal comfort, and energy efficiency of the building. The main goal of this study is to investigate whether a slight leak due to an unsealing of the joint by ~3 mm affects the thermal insulation of the connection. The study puts forward research hypotheses that contains a statement, which is used to analyze: St 1, high temperatures on the inner surface of the sandwich panel facing, reaching 100 °C, which does not cause damage to the joints between adjacent roof panels, and St 2, a slight unsealing of the joint by ~3 mm that does not affect the increase in the thermal transmittance and heat flux density in a roof partition constructed of sandwich panels.

## 2. Materials and Methods

The structure of the roof partition may be damaged, e.g., as a result of the thermal stresses that occur during a sudden and uncontrolled increase in temperature. As a result, the thermal insulation of the partition may be significantly reduced. In such situations, it is necessary to carry out a diagnosis of the roof based on macroscopic and in situ analytical and numerical methods.

The choice of research methods was dictated by the need to conduct analyses in an existing building, where it was important to use non-destructive methods. Infrared thermography IR was selected to assess the temperature distribution on the surface of the roof panels, as it allows for non-contact recording of temperature differences on the surfaces under test. Roof damage diagnostics were performed using the macroscopic method in order to determine, for example, the condition of the PIR foam core. Numerical models were created using the finite element method to visualize the geometry of the joint and precisely model heat flow. The results obtained were additionally compared with calculations based on harmonized European procedures, which allowed for an assessment of compliance with applicable standards.

### 2.1. Subject of Research

**Figure 2:**
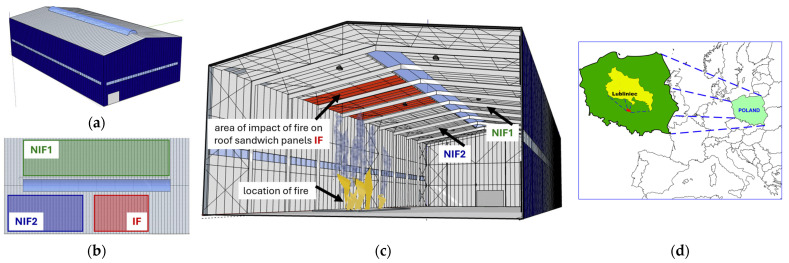
Subject of the study: (**a**) perspective view of the industrial hall with the roofing made of sandwich panels; (**b**) roof plan with a marked fire impact area (IF) and areas without fire impact (NIF1, NIF2); (**c**) a sketch of the interior of the hall, showing the location of the fire; and (**d**) map with location production hall in the Silesian Voivodeship, Southern Poland, Central Europe.

The assessment of the reaction to high temperatures was carried out for the roof of a production hall (Figure 2a) with a steel structure located in the Silesian Voivodeship, Southern Poland, Central Europe (Figure 2d). The hall has dimensions of 32 × 61 m and a height of 17.8 m. The roof covering of the hall, with an area of 1980 m^2^, was made of sandwich panels with a PIR foam core with a density of 40 kg/m^3^ and has a slope angle of 9°. The sandwich panels used in the building are commercial and widely available materials with thermal conductivity and mechanical properties declared by the manufacturer as compliant with standards [2,3,21]. The roof was installed in accordance with the manufacturer’s specifications. A single roof panel consists of an outer and inner facing made of S250GD galvanized steel sheet with a thickness *t_n_* of 0.5 mm and a core with a nominal thickness *d_c_* of 120 mm (Figure 1). The panel manufacturer recommends that the width of the joint between adjacent panels *wj* be 1–4 mm (detailed in Figure 1). For the facing colors, the external facing is in silver and the internal facing is in white. The effective width of a single panel is 1 m and its weight is 14.07 kg/m^2^. The heat transfer coefficient of the panel *U_d,S_* guaranteed by the manufacturer is 0.190 W/(m^2^·K) (taking into account the influence of mechanical connectors). The experimental procedure can be repeated for the tested material, and the results obtained in the article have direct practical significance because they can be applied to solutions used in existing implementations.

The assessment of the fire behavior of sandwich panels is determined in accordance with ISO 13784-1:2014 [41]. The technical parameters relating to the fire safety of the roof panel are as follows:

- Fire resistance up to REI 20 and up to RE 30;
- Roof resistance to external fire B_ROOF_ (t1);
- Reaction to fire B-s1,d0.

Six exhaust fans with 0.5 × 0.5 × 0.2 m Polyvinyl Chloride (PVC) collectors were installed in the roof panels. A fire broke out in the existing production hall (Figure 2c) as a result of the uncontrolled spread of fire arisen during the drying process of small-sized components in industrial production. The incident resulted in uncontrolled heating of the internal air. Thanks to a quick response to the incident by extinguishing the fire and turning on the exhaust fans, the fire did not spread to the entire facility. The fire lasted less than an hour and grew to a height of no more than 3 m above the floor level. Fire blankets were used during the firefighting operation, which slowed down the burning process and reduced the rate of heat release, but their improper use caused the flames to partially spread beyond the protected area. The fire was completely extinguished within 1 h. Immediately after the incident, temperature measurements were taken using a pyrometer. In the area exposed to direct fire, which is marked in Figure 2b,c as IF, the *T_si_* temperature on the surface of the internal facing was 100 °C. In the remaining areas of the roof, the temperature *T_si_* on the inner surface of the partition reached a value of up to 50 °C, which is consistent with standard roof operation. According to the manufacturer’s recommendations, the constant temperature on the surface of the panels should not exceed +60 °C. Areas beyond the fire impact zone are marked as NIF1 and NIF2.

### 2.2. Research Procedures

The calculation of heat loss through the roof partition was performed based on harmonized European standards. In accordance with European procedures, the heat transfer coefficient *U* is determined in accordance with the general method described in ISO 6946:2017 Building components and building elements—Thermal resistance and thermal transmittance—Calculation methods [42]. In the case of a partition with air voids, the heat transfer coefficient must be corrected. The general formula for the corrected thermal transmittance *U_c_* then takes the following form:

(1) $$U_{c}=\;U{+\Delta U}_{g}$$

where

*U*—thermal transmittance coefficient W/(m^2^·K),
Δ*U_g_*—with a correction for air voids W/(m^2^·K). In this research, a correction level of 1 was adopted due to the occurrence of air voids, where Δ*U″* is 0.01.

The thermal transmittance coefficient for homogeneous partitions was determined according to:

(2) $$U=\frac{1}{R_{si}{+R}_{1}{+R}_{2}{+...+R}_{n}{+R}_{se}}$$

where

*R*_1_, *R*_2_,…*R_n_*—design thermal resistance of each layer (m^2^·K)/W,
*R_si_*, *R_se_*—internal (*i*) and external (*e*) surface resistance (m^2^·K)/W.

The correction factor Δ*U_g_* should be found based on the expression:

(3) $$\Delta U_{g}=\Delta U^{\prime \prime }\;\cdot {\left(\frac{R_{1g}}{R_{si}{+R}_{1}{+R}_{2}{+...+R}_{n}{+R}_{se}}\right)}^{2}$$

where

*R*_1*g*_—thermal resistance of the layer that contains the gaps (m^2^·K)/W.

However, in the case of roof sandwich panels, the thermal transmittance coefficient *U_d,S_* should be determined in accordance with the standard EN 14509:2013 [21], which is dedicated to these products, where it is found using two methods. The first method considers the geometry of the panel profile and the thermal impact of the longitudinal joint, while the second is a simplified method that ignores the impact of the profiled surfaces and the linear thermal transmittance coefficient is assumed according to the general type of joint (*f_joint_*). In this study, the simplified method was used for the analysis, where *U_d,S_* was determined according to the formula:

(4) $$U_{d,S}=\frac{1}{R_{si}+\frac{d_{c}}{\lambda_{c}}{+R}_{se}}\;\cdot \;\left(1+f_{joint}\cdot \frac{1.0}{B}\right)$$

where

*d_c_*—nominal thickness of core (m),
*λ_c_*—declared thermal conductivity of the core W/(m·K),
*B*—width of panel *B* = 1.1 m,
*f_joint_*—where *f_joint_* is the linear thermal transmittance coefficient of the joint W/(m·K). Joint type IV was adopted in accordance with Table A4 in a standard [21], calculated for a joint distance of 1.0 m and the design thickness of the slab *d_d_* (Figure 1), the value of *f_joint_* = 0.019.

The design thickness *d_d_* of the roof sandwich panel is the total thickness of the facings and the core, determined in accordance with:

(5) $$d_{d}=\left(t_{ni}+\;d_{c}+t_{ne}\right)$$

where

*t_ni_*, *t_ne_*—nominal thickness of the internal (*i*) and external (*e*) facing (m).

The heat flux densities *q* W/m^2^ was determined according to the formula [43]:

(6) $$q=U\;\cdot \;\left(T_{i}-T_{e}\right)$$

where

*T_i_*, *T_e_*—internal (*i*) and external (*e*) temperature (°C).

### 2.3. Macroscopic Tests

**Figure 3:**
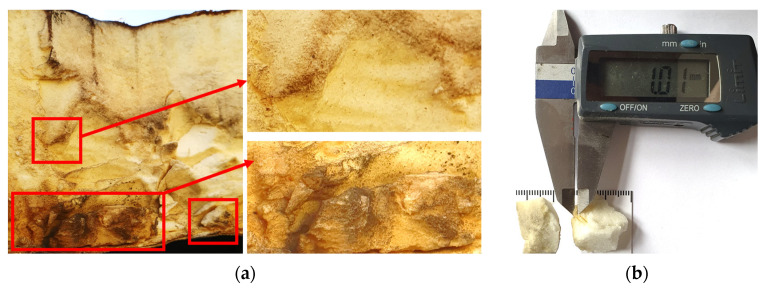
PIR foam at the fan installation site in the high temperature zone: (**a**) cross-section through the board with red markings indicating local surface melting of the core; and (**b**) sample taken from the high temperature zone.

An assessment of the technical condition of the roofing, with particular emphasis on the IF zone exposed to elevated temperatures, was carried out during an on-site inspection on 5 December 2024. In order to determine the extent of the damage, it was necessary to dismantle the fans in the IF zone. Based on a visual inspection of the cross-section of the roof panel, no significant defects in the core or deformation of the panel facing were found. The high temperature at the point where the core connects to the hot side surface of the exhaust fan caused slight melting of the PIR foam, as shown in Figure 3a. The assessment of the core samples taken from the area under investigation showed that the thickness of the damage is approximately 1.01 mm (Figure 3b) and do not affect the working of the whole component. Similar conclusions were arrived at by researchers in paper [35], where a fragment of a charred PIR foam sample was examined. It was found ‘mass did not suffer any deformations or a considerable loss of material, retaining its original feature almost intact’ [35] (p. 9493).

**Figure 4:**
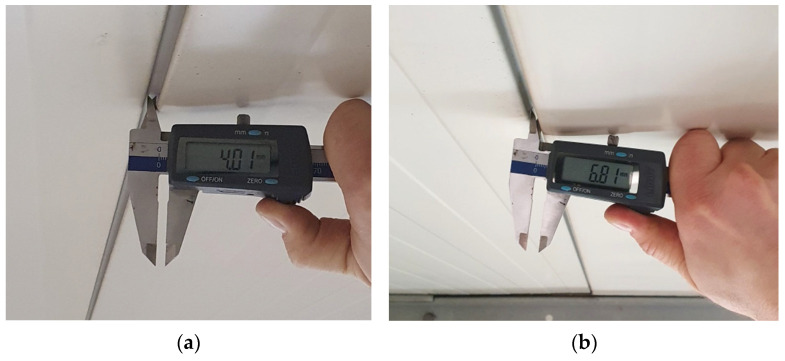
Measurement of the width of the lower part of the *wj* joint: (**a**) NIF1 area in the middle of the panel span with a measurement value of 4.01 mm; and (**b**) IF area at the support with a measurement value of 6.81 mm (one of the panels slightly raised).

The roof diagnostics were supplemented with an examination of the continuity of the panel assembly. Even short-term exposure to high temperatures can cause deformation in the form of distortion or local buckling of the panel. Particular attention was paid to assessing the air-tightness of the joints between adjacent panels (Figure 1, marked 4). Measurements of the width of the lower part of the joints *wj* were taken using a caliper (Figure 4).

The data obtained from measurements of the width of the lower part of the joint *wj* in the area exposed to high temperatures (IF) and the remaining part of the roof (areas NIF1 and NIF2), in accordance with Figure 2b, form the basis for further research and are given as follows:

- NIF1: *wj* ≤ 5.25 mm;
- NIF2: *wj* ≤ 4.89 mm;
- IF: *wj* ≤ 6.81 mm.

Some of the results exceed the maximum value of 4 mm recommended by the manufacturer (see Figure 1). A slight separation of the panels may have occurred during the installation of the panels, during the operation of the roof or as a result of short-term exposure to fire. It should be noted that, at the point where the highest result was obtained (i.e., 6.81 mm), one of the sandwich panels is located slightly higher than the adjacent panel. The study examined how widening the joint by ~3 mm (then *wj* = 6.81 mm) affected the thermal insulation of the connection.

### 2.4. In Situ Thermography Testing

Normally, the connection between two adjacent panels forms a small linear thermal bridge. However, uncontrolled unsealing of the joints can lead to a loss of thermal insulation and, as a result, to air infiltration inside the connection. This situation results in water vapor condensation and, thus, the escape of condensate from the joint, which contributes to, for example, moisture penetration of the partition. The detection of the unsealing within the joints was supported by in situ tests, on the basis of which the thermal parameter *T_si_*, determined directly on the internal surface of the sandwich panels, was examined.

**Figure 5:**
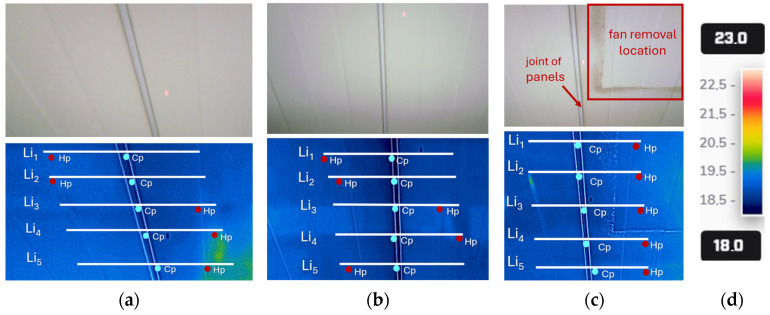
Measurements with a thermal imaging camera of the internal facing of the sandwich roof panels (red dot indicates maximum temperature and blue dot indicates minimum temperature): (**a**) NIF1 zone; (**b**) NIF2 zone; (**c**) IF high temperature impact zone, location next to the dismantled fan; and (**d**) legend of the color scale for a thermogram.

Thermography was used to assess the temperature distribution on the tested surface of the panels, which is commonly used to detect temperature values and anomalies on building envelope surfaces [44,45]. This method enables the creation of thermal images that visualize temperature differences on the tested surfaces, i.e., thermograms. The latter are created by presenting the temperature distribution in the form of colored isotherms, each of which represents a temperature range specified on a color scale. In situ tests were carried out using a thermal imaging device—a FLIR E95 camera with an LCD monitor with a temperature measurement range from −20 to 1500 °C and a resolution of 464 × 348 (161,472 pixels). The thermal sensitivity for the lens is 42° × 32° < 30 m·K and the spectral sensitivity range is 7.5–14 μm. This device allows for real-time observation and analysis of the measurements. All the thermograms are presented in multi-spectral dynamic imaging (MSX) technology, which means that the contours of the real image are superimposed on the thermogram using a color scale from 18 to 23 °C (Figure 5).

The main objective of the thermogram analysis was to examine whether there were significant discrepancies of several degrees Celsius in the *T_si_* temperature values measured within the joints. In addition, the analysis aimed to identify any anomalies in the temperature field distribution that could indicate damage to the joint. The thermograms were made in accordance with Figure 2 for the following areas:

- NIF1 and NIF2 outside the area affected by high temperatures during the fire;
- IF for the area directly above the fire.

Figure 5 shows selected thermograms for the analyzed zones, which are compared with a photograph of the measurement site. The thermal images for zones NIF1 and NIF2 are representative models, and the obtained temperature field distribution values were used as a reference for further research. The area of the joints between adjacent plates was assessed. In each case, five lines marked as Li were introduced, and then the temperature distribution along the straight lines was analyzed. The coldest point (Cp) is indicated by a blue dot and the hottest point (Hp) is indicated by a red dot.

### 2.5. Methodology of the Numerical Analysis

**Figure 6:**
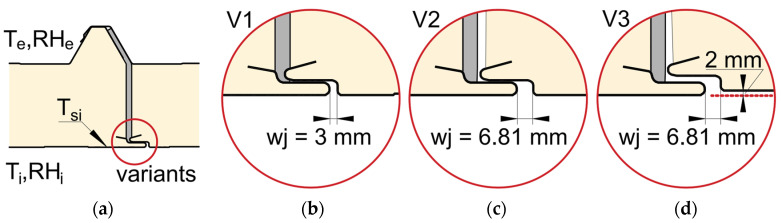
Numerical research models for different variants of the lower part of the joint: (**a**) model geometry; (**b**) V1, representative model *wj* = 3 mm; (**c**) V2, horizontal gap between panels *wj* = 6.81 mm; (**d**) V3, gap between adjacent panels *wj* = 6.81 mm with a slight elevation of one panel by ~2 mm.

The temperature field within the joint was modeled using the finite element method (FEM) [46,47] and modeling a sandwich panel joint according to the assumptions of the standard ISO 10211:2017 [48]. Research models for various joint unsealing variants (Figure 6) were created in the form of two-dimensional numerical models of the roof partition using THERM 7.6 software [49], this method is commonly used [50,51]. Under specified environmental boundary conditions for air temperature and relative humidity, the distribution of the temperature field and heat flux was analyzed for the test models, and the thermal transmittance coefficient was determined.

The following calculation variants were constructed:

- Variant V1 represents a representative model constructed in accordance with the manufacturer’s recommendations with *wj* = 3 mm (Figure 6b);
- Variant V2: this model presents an extreme situation regarding the horizontal gap between adjacent slabs with *wj* = 6.81 mm (Figure 6c);
- Variant V3: this model presents an extreme situation in which the horizontal gap between adjacent slabs with *wj* = 6.81 mm and slight lifting of the slab by ~2 mm (Figure 4b and Figure 6d).

The material data for the joint between two adjacent sandwich roof panels was adopted in accordance with the manufacturer’s guidelines. The thermal parameters of the sandwich roof panel components are summarized in Table 1.

The analysis of the temperature distribution in the partition was performed for the environmental conditions prevailing on the day of the on-site inspection. In addition, for external partitions, it is necessary to determine the permissible thermal load caused by the temperature difference between the internal and external sides of the partition. In most cases, manufacturers of roof sandwich panels specify the permissible thermal load caused by the temperature difference between the facing layers. For the product analyzed in this study, the manufacturer allows for a temperature difference Δ*T* of 50 °C.

The following boundary conditions were assumed for the calculations:

(1) event occurrence—the temperature is external *T_e_* = −2 °C and internal *T_i_* = 100 °C, temperature difference (*T_i_* − *T_e_*) is Δ*T* = 102 °C, and the relative air humidity is external *RH_e_* = 70% and internal *RH_i_* = 30%,
(2) in situ tests—the temperature is external *T_e_* = 2 °C and internal *T_i_* = 19 °C, temperature difference Δ*T* = 17 °C, and the relative air humidity is external *RH_e_* = 77% and internal *RH_i_* = 60%.

The surface thermal resistance values were selected in accordance with ISO 6946 [42] for upward heat flow, where *R_si_* = 0.10 (m^2^·K)/W and *R_se_* = 0.04 (m^2^·K)/W. The heat transfer coefficients on the surfaces are *h_i_* = 10 W/(m^2^·K) and *h_e_* = 25 W/(m^2^·K). The edge surfaces in the variants V of the test models were assumed to be adiabatic, while the other parameters, e.g., temperature, were in accordance with the boundary conditions described above.

## 3. Results and Discussion

### 3.1. Measurement Results

**Figure 7:**
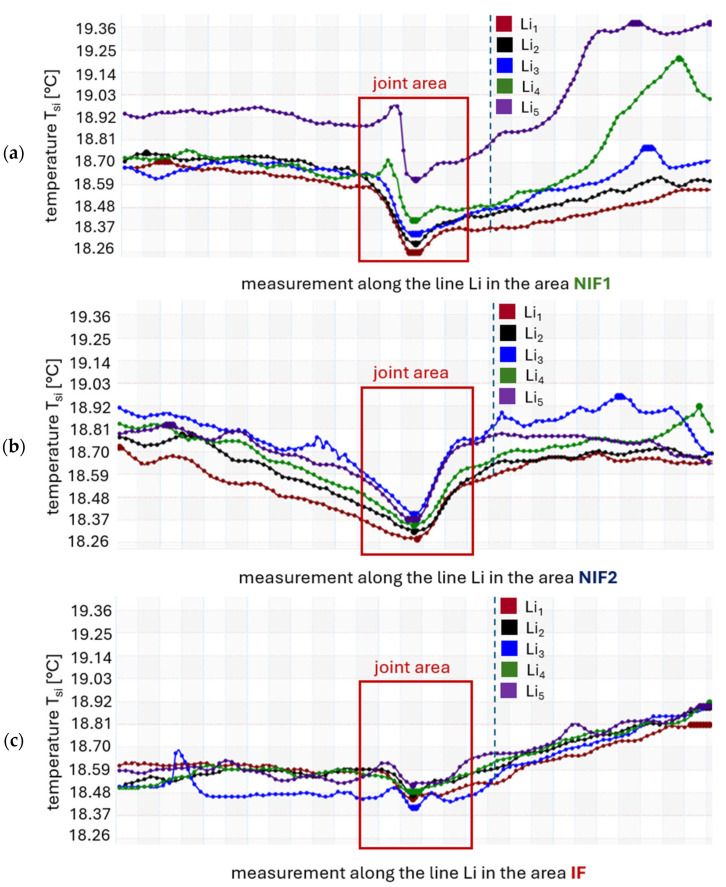
Graphical representation of the thermographic survey results: (**a**) NIF1 roof area; (**b**) NIF2 roof area; and (**c**) roof area above the IF fire site.

The results of thermographic tests show *T_si_* values for different areas of the roof, as shown in Figure 2. Based on the temperature distribution along the Li lines introduced in the thermograms (Figure 5), the results obtained in the area exposed to high temperatures, IF, were compared with the roofing areas without direct exposure to fire, NIF1 and NIF2. The internal surface of two adjacent panels located within the joint was analyzed, as the air-tightness of the panel connection is the main subject of consideration in this work. The basic signal indicating possible joint sealing failure will be significant differences in the temperature distribution for the analyzed zones. A graphical representation of the thermographic test results is presented in Figure 7.

For the NIF1 roofing zone, temperatures ranging from 18.26 to 19.38 °C were obtained and, for the NIF2 zone, temperatures ranging from 18.27 to 18.96 °C (Figure 7) arose. In turn, temperatures ranging from 18.41 to 18.90 °C were recorded in the IF zone. The maximum temperature difference between the IF and NIF1 zones is 0.97 °C, and between the IF and NIF2 zones is 0.55 °C. The temperature values obtained in the area directly above the fire do not differ significantly from those obtained in the zones outside the fire’s reach. The differences are minimal and do not exceed 1 °C.

Furthermore, the temperature distributions for lines Li_1_ to Li_5_ in individual zones are similar to each other and have a similar graphical distribution. Although there are some deviations on the right-hand side of lines Li_4_ and Li_5_ in relation to lines Li_1_ to Li_3_ in zone NIF1, the maximum difference between Li_5_ and Li_1_ is 0.79 °C. However, this difference is due to light reflected from the lighting on the inner surface of the panel, as seen in Figure 5a. The thermal imaging test also did not reveal any significant differences in temperature values within the joints and the central area of the roof panel, with the maximum temperature difference not exceeding 0.77 °C for the NIF1 zone, 0.69 °C for NIF2, and 0.5 °C for the IF zone.

Moreover, temperature measurements at selected locations of the roof facing have similar values and range from 19.38 to 18.26 °C, thus differing by a maximum of 1.12 °C, which indicates a correct temperature distribution across the entire roof surface. The maximum difference between the in situ results does not exceed 0.97 °C. The results for the minimum, maximum, average temperature, and standard deviation obtained from the thermographic tests are summarized in Table 2.

#### Validation of the Obtained Results

A preliminary assessment of the temperature distribution within the joint showed that the differences between the temperature values in different zones of the roofing are not significant, amounting to a maximum of 1.12 °C. This indicates that temperatures of the order of 100 °C on the internal surface of the facing did not adversely affect the joints between the panels. Therefore, it can be concluded that the hypothesis St 1 set forth in this paper is true.

**Figure 8:**
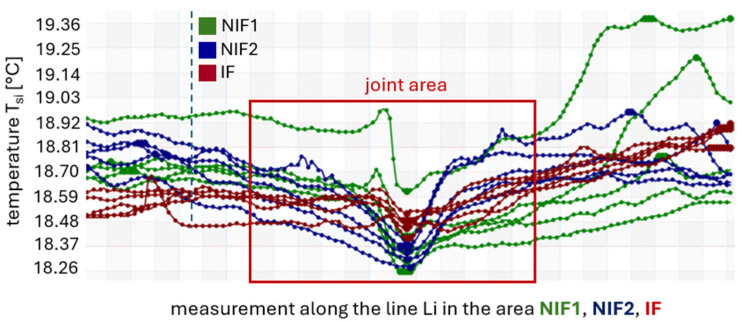
Graphical representation of the *T_si_* temperature results for areas around the joint that were statistically evaluated.

To validate the obtained results and confirm the St1 hypothesis, a precise evaluation of the collected data from the obtained temperature values was carried out. The area of analysis was narrowed to the strict perimeter of the joint, marked with a red rectangle in Figure 8.

The results from the *T_si_* temperature measurements recorded for each area were analyzed. This activity aimed to compare and evaluate the distribution of the data obtained in the IF area in relation to the NIF1 and NIF2 areas. The evaluation included the following:

- Temperature values, including the mean, minimum, maximum, and median;
- Standard deviation and standard error;
- Asymmetry of distribution.

When the mean values are compared with each other, they have similar values in all areas, with a range of 18.55–18.58 °C, while the medians range across 18.56–18.61 °C. The data in the IF area, NIF1, and NIF2 have a distribution with a left-handed asymmetry because the mean value is less than the median value. In the IF area, the minimum value of the *T_si_* temperature is 0.15 °C higher than in the NIF1 area and 0.14 °C higher than in the NIF2 area. In contrast, the maximum value in the IF area of the *T_si_* temperature is 0.29 °C lower compared to the NIF1 area and 0.2 °C lower relative to the NIF2 area. The differences in the *T_si_* temperature values are negligible and vary little between the analyzed sites.

The standard deviation is very low in all cases. A maximum value of 0.172 was obtained for the NIF1 area, which shows that the temperature values obtained are stable, predictable, and clustered around the mean. The standard error for the data in all areas is minimal and is 0.005 for IF, 0.014 for NIF1, and 0.012 for NIF2. These results confirm that the data are consistent and not very scattered, so the sample mean effectively reflects the average *T_si_* joint temperature for the whole roof. The temperatures for the entire roofing area are within the range obtained.

**Figure 9:**
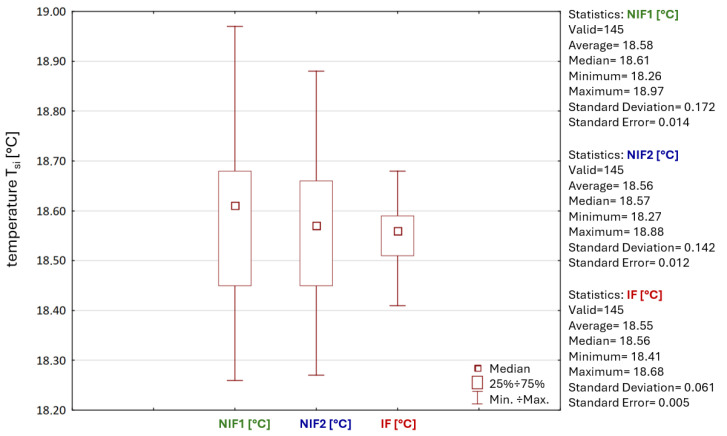
Box-plot diagram for the comparison of the distribution of the *T_si_* temperature data for the analyzed canopy areas.

In summary, the range of variation in the *T_si_* temperature within and between areas is small. It can, therefore, be concluded that there are no anomalous *T_si_* temperature values in the IF zone, which could indicate damage to the panel joint as a result of an extremely high temperature (*T_si_* = 100 °C). The results of the analyses of the distribution of temperature values for the individual areas are summarized in Figure 9.

### 3.2. Results of Numerical Analyses

**Figure 10:**
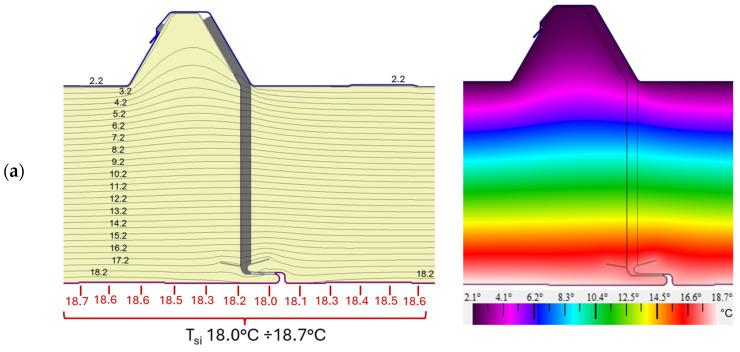

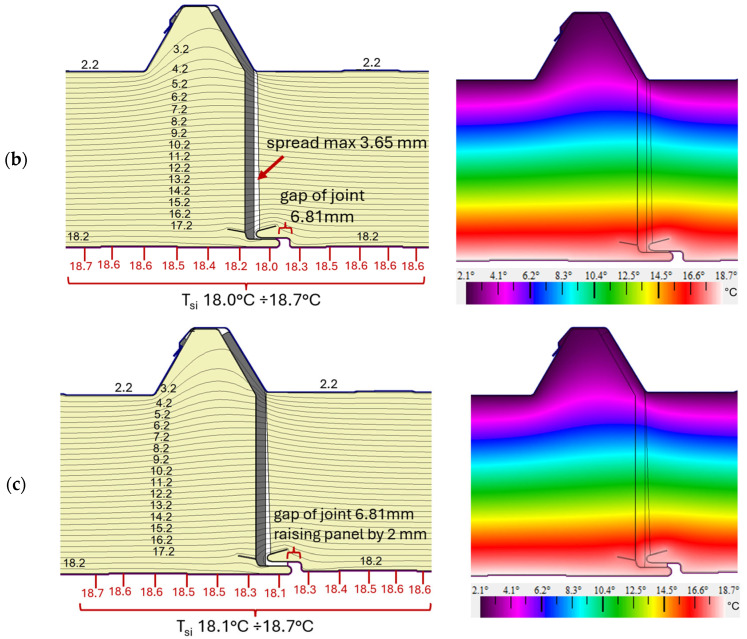
Distribution of isotherms (**right**) and distribution of the color-scale temperature field (**left**) for the panel joint area: (**a**) V1, representative model where *wj* = 3 mm; (**b**) V2, a test model where *wj* = 6.81 mm; and (**c**) V3, test model where *wj* = 6.81 mm with a slight uplift of one panel.

Numerical models were used to determine what effect a slight unsealing of the joint (~3 mm) has on the heat flow rate or temperature field distribution. A test model in which the joint was made according to the manufacturer’s recommendations (Figure 1 and Figure 6b), where the width of the lower part of the joint *wj* = 3 mm, was used as a representative model. The analysis was carried out for the environmental conditions of the in situ test day. The results are presented in the form of a distribution of isotherms, from which it is possible to identify isotherms that are closer to each other, which may be indicative of thermal stresses or structural problems. In turn, the results of the color infrared temperature field distribution indicate the presence of extreme temperature gradients (Figure 10).

The obtained *T_si_* temperatures in the numerical models on the surface of the internal facing validate the results obtained from infrared temperature measurements around the joints (18.26–18.97 °C). The results are consistent with the thermographic measurements and the *T_si_* temperature values for test models V1, V2, and V3 range from 18.0 to 18.7 °C, as presented in Table 3. The maximum difference between the methods does not exceed 0.3–0.4 °C for averages and 0.26–0.27 °C for extreme values. Both methods record similar ranges of minimum and maximum temperatures, which confirms the reliability of numerical models. Thus, the obtained results are very close and satisfactory, considering that the temperature measurements using thermography are affected by many external factors such as light reflected from the surface, imperfections in the mounting of the panels, and others. Numerical models can be considered a reliable validation and prediction tool in the thermal analysis of roof panels.

From analyzing the distribution of the isotherms and the distribution of the temperature field for variants V1 and V2, it can be seen that a slight unsealing of the joint by 2.81 mm in V2 does not result in a decrease in temperature values compared with the representative model V1. Similarly, in variant V3, where in addition the panel is slightly raised by 2 mm, the temperature field distribution is similar to that for variant V1. This means that the slight separation of the panels with a maximum value of *wj* = 6.81 mm, measured during macroscopic testing (Figure 4b), does not adversely affect the temperature distribution on the internal facing surface.

For the prepared test models of the roof partition constructed from the components listed in Table 1, the thermal transmittance *U_V_*_1_ = 0.196 W/(m^2^·K), *U_V_*_2_ = 0.187 W/(m^2^·K), and *U_V_*_3_ = 0.189 W/(m^2^·K) were obtained, without considering the influence of mechanical fasteners. It can be seen that the geometry of the type of joint solution discussed in this article means that increasing the joint width *wj* improves the thermal insulation of the partition slightly. The air layer trapped between the upper lining of the panel in the V2 and V3 variants and the specially profiled shape of the lower part of the joint is a good thermal insulator. The resulting gap between the board core and the PU expansion gasket in the test models has a maximum width of 3.7 mm. The gap is thin and there is no significant air circulation. Air movement is restricted and does not flow freely between the inside of the gap and the surroundings. Thus, any heat transfer by air movement (i.e., convection) is minimal.

When analyzing the V2 and V3 solutions, it should be noted that the horizontal separation of the panels in the V2 variant impedes air movement in the resulting joint (Figure 10b). The shaping of the lower part of the joint, namely, the bending of the internal facings of the panel, ensures that the width of the gap inlet is small—in the V2 test model, it is 0.5 mm. In the case of the V3 solution, where one of the panels is slightly raised relative to the other (by 2 mm), the joint inlet is increased to 2.5 mm (Figure 10c). In this situation, the thermal transmittance increases compared with V2. It is important that the installation of the panels is carried out with the internal facings level with the adjacent panels to ensure that little or no air circulation is maintained. Significantly raising the sandwich panel results in convective movement of air inside the joint, which consequently worsens the insulation properties of the joint and may lead to condensation inside the joint. Another worrying fact is that PIR foam can emit toxic gases when exposed to high temperatures, as analyzed in [26,27,28]. Researchers in [37] point to a reduction in joint stiffness at elevated temperatures for panels with a PIR foam core, and it is possible that the slight lifting of the panel was caused by the effect of high temperature. Therefore, the situations outlined in V3 need to be controlled. The values of the thermal transmittances obtained via the varying methods are summarized in Table 4. The maximum value of the width of the gap, *wj* = 6.81 mm, does not adversely affect the thermal protection status of the partition.

The *U_d,S_* thermal transmittance from the manufacturer has been calculated by taking into account the linear thermal bridge that occurs at the interface between the sandwich panels and the point thermal bridges, which arise where the panels are attached by fasteners to the supporting structure. The thermal transmittances obtained (Table 4) meet the national thermal insulation requirements [5] for maximum values for roofs.

Three independent calculation methods were used: ISO 6946:2017, EN 14509:2013, and the THERM 7.6 numerical method. The obtained thermal transmittance values (Table 4) show consistency within 0.01 W/(m^2^·K), which confirms their mutual reliability. In addition, these values are consistent with the manufacturer’s data, taking into account the influence of linear and point bridges. The numerical results, validated by thermographic measurements (Table 3), are a reliable tool for predicting and evaluating the thermal behavior of roof panels joints and confirm the correctness of the conduction model in the joint.

**Figure 11:**
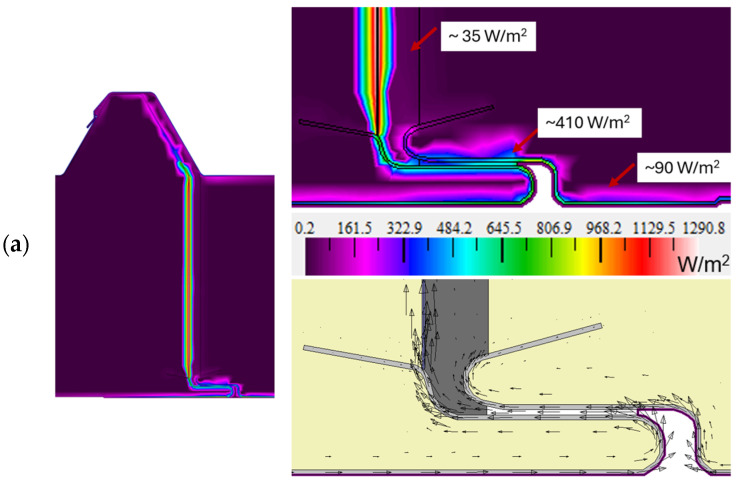

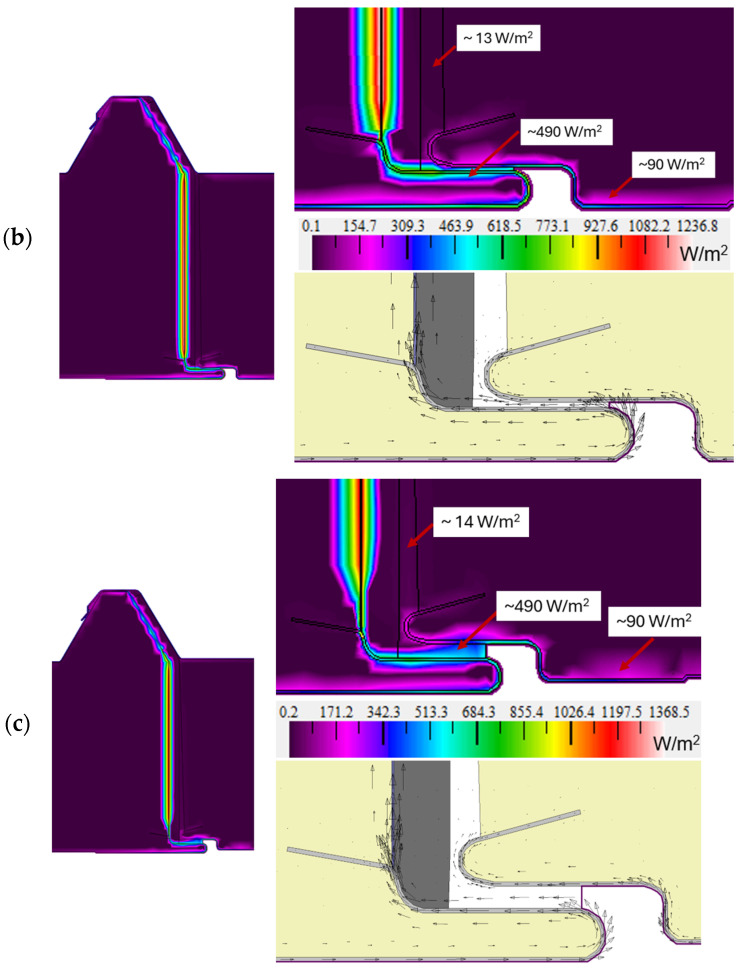
Heat flux density distribution within the junction of the two adjacent color-scale panels. On the right-hand side is a view of the whole joint and on the left-hand side is the lower part of the joint for the test models: (**a**) representative V1; (**b**) V2; and (**c**) V3.

The test model V1, as a representative connector, and V2 and V3 were assessed for heat flux density distribution; the graphical results are presented in Figure 11. The heat flux is determined as a vector quantity based on the thermal conductivity and the local temperature gradient surrounding the connector according to Formula (6). The graphical representation, particularly at the lower part of the joint (Figure 11, right-hand side), indicates the amount and direction of heat flow determined from the heat flux vector (the direction of the arrow indicates the direction of flow). The heat flux density vectors are always directed perpendicular to the isotherms and have the same direction as the temperature gradient. The color representation, in turn, indicates the magnitude of the heat flux (purple indicates low flux and red indicates higher flux).

An analysis of the flux density of the representative model V1 (Figure 11a) shows that, throughout, it is very low in principle at around 13 W/m^2^ (dark purple areas). For the vast majority of the joint, it remains at around 35 W/m^2^. Only within the steel sheet facings does it assume a higher value of ~410 W/m^2^ and even ~800 W/m^2^. The highest values were obtained at the insertion point of the vapor barrier foil with an aluminum layer at ~1100 W/m^2^ (even up to ~1290 W/m^2^). This phenomenon is perfectly normal due to the high thermal conductivity of aluminum that, in combination with the insulating layer, acts as a good insulator and does not significantly affect heat loss through the envelope.

Similar results were obtained for variant V2 (Figure 11b), the value of the heat flux density in the resulting gap and the core of the panel being approximately 13 W/m^2^. As in the representative model, disturbances in the heat flux density distribution can be observed within the joint inlet (from ~400–800 W/m^2^) at the steel facing locations and at the vapor barrier film insertion location (values from ~1100 W/m^2^ to ~1230 W/m^2^). Analyzing the distribution of heat flux density within the joint for variant V3, similar results were obtained as for the other cases. The resulting gap has a value for the flux density of 14 W/m^2^. At the inlet to the joint, values of ~400–800 W/m^2^ were obtained.

In summary, the lower the value of the heat flux density, the better the thermal insulation of the roof envelope and, thus, the lower the thermal energy transfer. The presented visualization of the heat flux in Figure 11 showed that, in the analyzed areas, for the adopted solutions V1, V2, and V3, there is a low density of heat flux (a low thermal energy transfer), which indicates good thermal insulation for the joint. In all the variants, i.e., V1, V2, and V3, a low heat flux density q was obtained for the whole partition from 3.17 to 3.33 W/m^2^; the results are summarized in Table 5.

The hypothesis St 2 is true. However, it is important to watch out for situations where there will be very large separations between adjacent panels or significant lifting of panels relative to each other. If air circulates in the resulting gap, this situation affects the thermal and moisture protection. The results obtained are consistent with the findings of [40], where the authors drew similar conclusions when analyzing wall panel joints. Only a air-tightness joint guarantees protection against losses resulting from air infiltration inside the joint.

**Figure 12:**
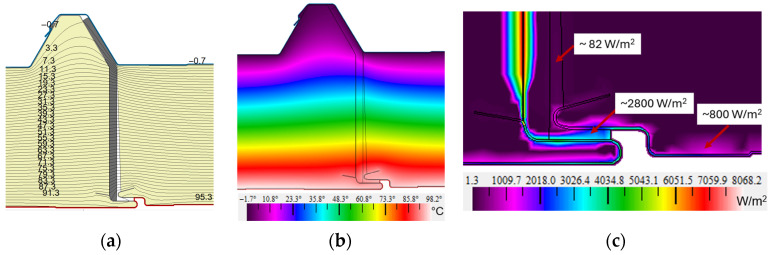
Results for variant V3 with a panel joint zone in a high temperature situation: (**a**) joint with isotherms plotted; (**b**) distribution of temperature field in a color scale; and (**c**) visualization of heat flux density in a color scale.

For the product analyzed in this paper, the manufacturer allows for a temperature difference Δ*T* of 50 °C. The roofing of the industrial hall was exposed to high temperatures for a short period of time during the incident described. The behavior of the roof sandwich panel was tested in the extreme case where the temperature difference Δ*T* was 102 °C. The results for variant V3 are presented in Figure 12. The values obtained for the temperature field distribution clearly showed that the resulting thermal load, does not cause any disturbance in the distribution.

In an analysis of the fire resistance of the structural elements, it is furthermore important to determine the heat flux density, which indicates the rate of heating of the partition. The heat flux visualization, as displayed in Figure 12c, showed that the heat flux density was ~82 W/m^2^ (dark purple areas) throughout. However, in the joint inlet area and the facing within the steel sheet facing, the flux density was critically high, from ~1200 to ~4800 W/m^2^. The highest values were obtained at the insertion position of the aluminum-lined vapor barrier foil, which were as high as ~8000 W/m^2^. Values of this level are obtained under specific conditions, e.g., in production halls with smelting furnaces. In the vast majority of the sections, the heat flux density is 19.237 W/m^2^ (Table 5) and within the joint area ~82 W/m^2^, which indicates good thermal insulation as well as meeting the requirements for fire insulation (I) and fire integrity (E) in the extreme situation.

**Figure 13:**
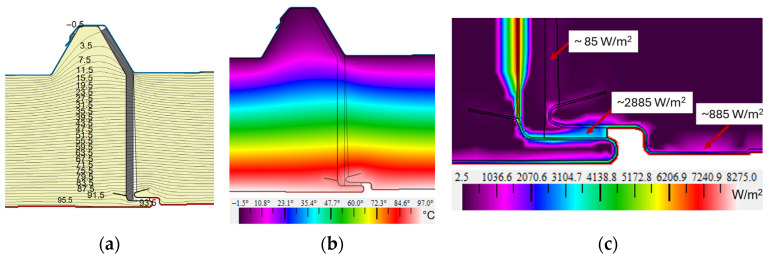
Results for variant V3, with high temperatures and increased thermal conductivity of PIR foam: (**a**) joint with isotherms plotted; (**b**) distribution of temperature field in a color scale; and (**c**) visualization of heat flux density in a color scale.

Researchers Wang and Foster, determined the relationship between temperature and thermal conductivity of PIR foam. They indicated that ‘the thermal conductivity of PIR foam increases exponentially with temperature’ [39] (p. 9) as well as for other materials for example, PUR foams and water [52,53]. At a temperature of 100 °C, the thermal conductivity for PIR foam is *λ* is 0.039 W/(m·K). The situation in which the thermal conductivity of PIR foam increases was therefore investigated. For comparison purposes, the analysis was carried out for variant V3 and a graphical representation of the results is presented in Figure 13.

The results obtained from the numerical modeling for the *U*-value of the thermal transmittance are 0.299 W/(m^2^·K) and the heat flux density q for the whole sandwich panel is 30.544 W/m^2^. These results indicate that the envelope exhibited significantly poorer thermal insulation conditions at the time of the fire. The thermal transmittance increased by 57% and the heat flux density by 59% for the V3 variant joint solution. However, even in this case, short-term exposure to high temperatures does not pose a danger to the use of the partition. Similar findings were reported by Makaveckas et al. [17], who pointed to the problem of a 15% increase in the thermal conductivity of PIR foam at high temperatures of +70 °C.

### 3.3. The Limitations of Study and Research Perspectives

The analysis of the impact of high temperatures caused by fire on self-supporting roof panels was carried out in an existing building, which is a rare situation and differs from tests conducted under controlled conditions in a laboratory. These tests were subject to certain conditions and limitations. Considerations were made only for one type of commonly used joint solution, without the possibility of comparing it with solutions proposed by other manufacturers. In addition, the core material for PIR foam was analyzed; it would undoubtedly be interesting to include other core materials in the analyses, e.g., PUR foam or EPS polystyrene. The experiment was conducted in one location and under specific climatic conditions without the possibility of long-term observation.

The limitations of the research conducted outline the directions for further research. The main objective in this area will be to conduct laboratory tests in a test chamber and to evaluate the long-term thermal behavior of the panels in various climatic and operating conditions. A good alternative would be to conduct a microstructure analysis for a more detailed assessment of the fatigue properties of the materials. This will allow for a direct comparison of the calculated values with the results of experimental measurements. Extending the research in the field of analysis to other types of geometric joint solutions and the use of different materials for the core will allow for a more complete understanding of heat transfer mechanisms.

The observations made during the research allow us to formulate practical recommendations. The correct installation carried out by a qualified company in accordance with the manufacturer’s requirements and technical documentation (Figure 1) ensured air-tightness and resistance to high temperatures. The internal linings should be kept level and the width *wj* of the lower part of the joint should be checked. It is recommended to use appropriate polyurethane (PU) seals, which improve air-tightness and reduce the formation of thermal bridges. Any negligence in the correct execution of the connection may result in a deterioration of the energy parameters of the entire partition.

## 4. Conclusions

The correct installation of the roofing made of sandwich panels on the basis of the manufacturer’s guidelines and in accordance with the principles of technical knowledge contributed to the creation of an airtight and heat-resistant partition. The tests did not identify any damage to the structure relating to the occurrence of excessive heating of the air inside the hall, such as thermal deformation of a structural element, loosening of mechanical fasteners, buckling, or bulging of the roof panels. The core of the roof panel at the fan location has locally occurring melting of the PIR foam, but the thickness of this damage (approximately 1 mm) does not represent a hazard and does not require remedial action.

In this paper, a series of analyses were carried out to evaluate the *T_si_* temperature on the surface of the inner lining of roof sandwich panels, particularly within the joint. The effect of the identified slight unsealing of the joint by ~3 mm on the thermal insulation behavior of the joint was determined. The main results and conclusions of the study are as follows:

1. The obtained *T_si_* temperature values in the IF area exposed to high temperatures do not differ significantly (differences do not exceed a value of 1 °C) from the temperatures obtained in the zones (NIF1 and NIF2) outside the high temperatures. On the other hand, the difference in the *T_si_* temperature values between the joints and the center of the roof slab does not exceed 0.77 °C for the NIF1 zone, 0.69 °C for NIF2, and 0.5 °C for the IF zone. Validation of the obtained results was achieved, in which the analyses were restricted to the strict perimeter of the joint; it was shown that there are no anomalous *T_si_* temperature values in the IF zone, which could be indicative of damage in the panel joint as a result of extremely high temperatures (*T_i_* = 100 °C).
2. Numerical test models with different variants of the solution of unsealing the lower part of the joint *wj* (V1 as a representative model with *wj* = 3 mm, V2 with *wj* = 6.81 mm, and V3 with *wj* = 6.81 mm for raising the plate by 2 mm) allowed for the analysis of the temperature field distribution in the partition, the determination of the heat flux density, and the discovery of the heat transfer coefficient. The obtained results show that a slight displacement of the panels with a value of *wj* = 6.81 mm does not adversely affect the temperature distribution *T_si_* on the internal surface of the facing. Similar findings were obtained by researchers Wang and Foster [39]. The thermal transmittance values are *U_V_*_1_ = 0.196 W/(m^2^·K), *U_V_*_2_ = 0.187 W/(m^2^·K), and *U_V_*_3_ = 0.189 W/(m^2^·K). The geometry of the lower part of the joint ensures that the joint is airtight even if the joint is slightly pulled apart.
3. Analysis of the heat flux density at a temperature difference of Δ*T* = 17 °C for the test models showed that, in all variants V1, V2, and V3, the low heat flux density q for the entire partition was obtained for 3.177–3.334 W/m^2^. In the case of the temperature difference Δ*T* = 102 °C, heat flux density q for the whole partition is from 19.237 to 20 W/m^2^, which proves that the thermal insulation is good and also meets the requirements of insulation (I) and fire resistance (E) in the extreme situation.

The evaluation of the response to high temperatures of the joint of roof sandwich panels with a PIR foam core presented in this paper confirms that these products are safe to use, which aligns with the tests carried out in ref. [35]. Short-term exposure to high temperatures of 100 °C did not cause significant damage to the facings, the core and, most importantly, the joint of the roof panels.

## Figures and Tables

**Table 1:** Compilation of material data forming the joint (Figure 1).

Layers of the Roof Sandwich Panel	Thickness (m)	*λ* (W/(m·K))
internal facing—galvanized steel sheet	0.0005	58
core—PIR foam	0.1200	0.022
vapor barrier film with aluminum layer	0.0003	20
polyurethane gasket	0.0050	0.045
butyl mass	0.0060	0.170
external facing—galvanized steel sheet	0.0005	58

**Table 2:** Compilation of the results for temperature *T_si_* measurements using thermography.

Area	Line	Temperature on the Internal Surface of the Roof Panel *T_si_* (°C)				
		Joint Area	Maximum	Minimum	Average	Standard Deviation
NIF1	Li_1_	18.26	18.70	18.26	18.52	0.12
	Li_2_	18.30	18.74	18.30	18.58	0.12
	Li_3_	18.35	18.77	18.35	18.60	0.11
	Li_4_	18.41	19.21	18.41	18.70	0.19
	Li_5_	18.61	19.38	18.61	18.98	0.21
NIF2	Li_1_	18.29	18.72	18.27	18.56	0.12
	Li_2_	18.33	18.78	18.31	18.61	0.13
	Li_3_	18.39	18.96	18.39	18.77	0.14
	Li_4_	18.34	18.92	18.34	18.67	0.14
	Li_5_	18.37	18.83	18.37	18.70	0.11
IF	Li_1_	18.45	18.81	18.45	18.62	0.09
	Li_2_	18.47	18.89	18.47	18.63	0.11
	Li_3_	18.41	18.90	18.41	18.57	0.14
	Li_4_	18.48	18.91	18.48	18.63	0.11
	Li_5_	18.51	18.89	18.51	18.65	0.11

**Table 3:** Validation of numerical predictions against experimental thermographic measurements of *T_si_* around the joints for the environmental conditions (i.e., with *T_e_* = 2 °C, *T_i_* = 19 °C, *RH_e_* = 77%, and *RH_i_* = 60%).

Method	Case	Temperature on the Internal Surface of the Roof Panel *T_si_* (°C)		
		Maximum	Minimum	Average
In situ	NIF1	18.97	18.26	18.58
	NIF2	18.88	18.27	18.56
	IF	18.68	18.41	18.55
Numerical method	V1	18.70	18.00	18.35
	V2	18.70	18.00	18.35
	V3	18.70	18.10	18.40

**Table 4:** Thermal transmittance of a roof sandwich panel obtained by different calculation methods.

*U * (W/(m^2^·K))			
Manufacturer	ISO 6946:2017 [42] (Formula 1)	EN 14509:2013 [21] (Formula 4)	Numerical Method THERM 7.6 [48]
*U_d,S_* = 0.190	*U_c_* = 0.188	*U_d,S_* = 0.182	*U*_*V*1_ = 0.196
			*U*_*V*2_ = 0.187
			*U*_*V*3_ = 0.189

**Table 5:** Summary of the results for the heat flow through the roof envelope for solution variants V1, V2, and V3.

Variants	Heat Flow (W)	Heat Flux *q* (W/m^2^)
in situ tests *T_i_* = 19 °C		
V1 representative model	3.907	3.334
V2	3.723	3.177
V3	3.751	3.205
event occurrence *T_i_* = 100 °C		
V1 representative model	23.441	20.002
V2	22.341	19.064
V3	22.544	19.237

## Data Availability

The original contributions presented in this study are included in the article. Further inquiries can be directed to the corresponding authors.
